# Health Physics (보건 물리학) in South Korea: Building a Research Community in a Post-Colonial Society, 1959–early 1970s

**DOI:** 10.1007/s00048-022-00334-x

**Published:** 2022-05-18

**Authors:** John P. DiMoia

**Affiliations:** Seoul National University (SNU), Seoul, Korea (Republic of)

**Keywords:** Nuclear, Health physics, Radiation medicine, Technical assistance, East Asia, Korea

## Abstract

This paper traces the diverse contexts of radiation protection from liberation in post-1945 South Korea to its professionalization by the early 1970s, using the emerging field of health physics (보건 물리학) as the focus. The Korean nuclear center, AERI (Atomic Energy Research Institute/한국 원자력 연구소, 1959), started two affiliates, RRIA (Radiation Research Institute for Agriculture/방사선 농업연구소, 1966) and RRIM (Radiation Research Institute for Medicine/방사선 의학연구소, 1963) in the early 1960s. In particular, RRIM emphasized the use of radiation within cancer research, especially the use of cobalt in treating patients. In this context, health physics initially took the form of “radiation medicine.”

With the two institutes returning to AERI’s fold in 1973, health physics took another turn: the second major subfield, following “radiation medicine,” invoked a broader conception of “radiation protection,” which now referred to a cluster of related interests, including the environment and the effects of industrialization.

Rather than a simple transfer of American and international models after 1953, Korean health physics reflects its origins in a post-colonial research setting, one more diverse than its “official” institutional context at AERI.

## The ROK (Republic of Korea) and the East Asian Context (1955–1959)

Recent literature addressing the distribution and use of radioisotopes has challenged the image of the atom in a variety of different ways (Creager 2013; Curry 2016; Hamblin 2009). In particular, this observation holds for the high degree of attention given to the goal of nuclear power, an aim associated with the “Atoms for Peace” program (DiMoia 2010). For nations in East and Southeast Asia, the shift in emphasis away from nuclear power offers a new perspective on many of these formative programs, helping to capture the original context in which research ambitions were set. Whether emerging from colonialism, war, or a similar set of challenging circumstances, there was frequently a conspicuous gap between the expectations of newly arriving experts, and those of Asian, African, and other actors, with this second group more familiar with the material circumstances of a given national or regional context (Osseo-Asare 2019). This article engages with the South Korean nuclear program in its first two decades (1959-late 1970s), focusing specifically on research community formation within health physics (**보건 물리학**), a central part of the ROK (Republic of Korea) program for much of the late 1950s that continued through the 1970s (KAERI 1979, 1989).

During and following the Korean War (1950–1953), South Korea became one of the world’s largest multi-lateral aid projects, the recipient of materials, technical knowledge, and generous sources of funding from as early as the fall of 1950 (UNKRA 1956). It is therefore not surprising that Korean nuclear ambitions have received a good deal of scholarly attention, typically linked to post-war developmental expectations (DiMoia 2010). In this context, the goal of South Korean rehabilitation formed an explicit contrast to that of its northern neighbor, transforming it into a showcase or model project situated in the midst of the Cold War competition (Hong 2015). Under these circumstances, alternate uses of the atom, such as for agriculture and radiation medicine, generally created fewer expectations, especially when they suggested a different or alternate path for shaping national ambitions. Moreover, these uses of the atom held close ties to previous history, hinting at Korea’s lengthy past within a Sino-centric East Asian region, as well as to Japanese empire (1910–1945) under colonialism (Kim 2018; Mizuno 2021).

This article tracks two related clusters of actors involved in the creation of a Korean health physics community from the time of the Korean War until the late 1970s. If health physics emerged in part with reference to AERI (Atomic Energy Research Institute, 한국 원자력 연구소 ), the research reactor and nuclear center created in conjunction with “Atoms for Peace,” it held an equally prominent role with respect to agriculture, medicine, and the environment well before the arrival of a formal atomic program. In refurbished hospitals, radiation medicine became the first field offering a possible future, as the handling and regulation of teletherapy units offered a critical context in which to engage with issues of use and standardization.^1^ In two types of settings—first, radiation medicine, then followed by environmental science—Koreans crafted a version of health physics suited to their particular developmental needs. Moreover, this trajectory suggests Korea’s setting within a broader East Asian research community, one in which Japan could re-emerge as a close partner after 1965, despite the colonial legacy.

### *Addressing Korean History*

This article examines these trends with the aim not to offer a position contrary to the goal of nuclear power, but rather, seeks to challenge an American-dominated institutional history for postwar Korea. Prior to the mid-1950s, South Korea’s networks of knowledge and exchange lay almost entirely within the East Asian region, and indeed, it was extremely uncommon to study abroad in either Europe or the United States prior to 1945 (Moon & Kim 2004). At the same time, it was difficult, but possible, for an elite few to study in Taisho or Showa Japan from the 1920s to the 1940s (Moon & Kim 2004; Kim 2007). Indeed, the second of these two options provided a powerful model of scientific practice for a number of colonial-era Koreans, with a limited few going on to pursue and achieve advanced degrees. By returning the emphasis to East Asia, this paper recognizes that there were a number of different ways in which the Korean program took on a reality distinct from that anticipated by its American and international patrons.

The historiography on the Korean atom program tends to regard the first two decades at AERI as transitional, preparation for what ultimately became a reactor-based program, with commercial power generation achieved by the late 1970s. Along these lines, the most widely cited article, Sang-hyun Kim and Sheila Jasanoff’s “Containing the Atom,” characterizes this initial period in terms of “atoms for national development,” with this label suggesting a teleology, a trajectory to follow (2009: 131). Similarly, the majority of Korean-language scholarship focuses primarily upon the question of power generation, with Kim-Seong Jun’s dissertation (2012) likely representing the best of the recent work. These approaches understandably focus upon the reactor as the most conspicuous object around which to structure a narrative. In doing so, however, the power question tends to minimize the role of prior history, especially during Japanese colonialism, as well as the surrounding regional context, and places Korean actors at the center of the narrative working in conjunction with their new American counterparts. By placing AERI at the center, this literature also excludes the multiple uses of radiation external to the official institutional context.

This post-war generation of emerging Koreans researchers received American consultants in health physics during the installation phases for “Atoms for Peace,” and maintained their own sub-section devoted to this specialty within AERI. However, the career trajectory of these Korean researchers was often different from that of their American counterparts, and the possible reasons informing this distinct character deserve attention. The senior personnel at AERI tended to be chemists and physical scientists prior to the early 1970s, as there were few or no nuclear engineers as such until that time. Similarly, those running the Korean version of health physics, such as Rho Chae-sik (노재식), head of AERI’s health physics for much of the period (1960–1973), most often trained in meteorology and environmental science, looking at pollutants and their effects upon the atmosphere. An end product shaped by a combination of contingency, need, and the industrial ambitions of a developing country, Korean health physics serves to emphasize the distinct historical and material conditions shaping the program, set in contrast to the baseline assumption of a nuclear program devoted to conjoined goals of power and reactor development. The ambition of nuclear power imposes a false coherence on a much more complicated set of origins, especially as Koreans worked out the challenging politics of determining their own post-colonial identity.

## Nuclear Resources and Formative Development

For the South Korean program, this revised frame makes a great deal more sense in context, as an energy-driven program would not become a priority until at least the mid to late 1960s, at which point it emerged only under very different political circumstances. Instead, much of the early nuclear program was shaped by two major factors: the recruitment of personnel, and the role of radioisotopes in fields such as medicine and agriculture, especially radiation therapy for cancer. For the first of these, the handful of staff in charge of the program at its inception were dominated by those with prior training in imperial Japan, including physicist Park Chul-Chae, the first head of AERI (KAERI 1979). Even as this small group dominated the early research trajectory, the site also became a much-valued selling point as a scientific center in the early 1960s, functioning in many respects as the rough equivalent of a national laboratory (KAERI 2009). Koreans returning from abroad, generally the US and Europe, to take university jobs sometimes received permission to affiliate with AERI so that they might hold a university teaching position while also using the site to network and to conduct their personal research. It bears mentioning that there were often tensions between these returnees and the older generation, especially for younger American-trained scientists and engineers.

The appeal of such a dual arrangement, combined teaching and research, was designed to attract these returning Koreans from abroad, especially those who had received scholarship opportunities during and in the aftermath of the war. The small, but vibrant scientific community was widely scattered at this time. Of the more than two hundred scientists fitting this category (237), a significant number chose to extend their overseas stays, completing advanced degrees and taking research fellowships or long-term positions (KAERI 2009: 53). Moreover, there was not a single destination favored for study, although the United States remained a top choice. Other popular sites included the UK, France, and West Germany, all sites with active programs in nuclear research (KAERI 2009). Collectively, this activity returned a diversity of approaches to moving the Korean program forward, sometimes leading to conflict with more senior staff. It also meant a disparity between the conditions the researchers encountered overseas and the more reduced material conditions of the Korean domestic program. After the return of these overseas group members, the legacy of American training has perhaps unsurprisingly received the bulk of scholarly attention.

The arrangement of allowing dual appointments, which encouraged the incremental development of a long-term research community, ran directly counter to much of the advice given to the Koreans under the original terms of “Atoms for Peace,” which urged a close focus around the reactor. In the three years since signing a bilateral agreement (1956–1959), members of AERI had selected a site (1957), broken ground, and declared the facility to be open for operations, although the reactor did not go critical until 1962. AERI also joined IAEA (August 1957), and would shortly begin receiving advice from its technical assistance missions (IAEA 1960). Working in close conjunction with advisors from the University of Michigan’s Phoenix Project for the reactor site, Korean scientists began to assemble a modest slate of research projects and ambitions, with the site functioning primarily as a scientific center and as a meeting point for the sharing of ideas. However, visiting consultants sent to check on the program, such as Leonard Reiffel, argued that the materials received had little to offer the local context: in the end Korea, remained a developing country.^2^ For his troubles, Reiffel found himself the recipient of heavy criticism from Michigan faculty members, who largely approved of the direction and scope of the Korean program.

What does this debate that took place about 1960, just as the research reactor was soon to go critical and at the height of staff recruitment, say about the provisional status of the Korean program? In Reiffel’s estimation, the danger lay in the possibility of pursuing an overly ambitious research program, one dominated by the ideals of “Atoms for Peace,” with little or insufficient attention to the actual needs of the Korean context. For their part, the Michigan faculty members responded strongly to his criticism, taking offense at the (perceived) personal nature of his remarks, and more importantly, at the scope of the program, which aimed at moving to the next level of reactor, with which they found few problems. At the same time, this debate underscores a fundamental tension with respect to a developing context like South Korea. As of 1960, the program taking shape closely resembled one authorized under more modest terms, that is, a program tied to work with radioisotopes for settings such as agriculture and medicine. If this earlier history has been forgotten in part, this is because of the perceived success of commercial nuclear energy, the dominant trend within the Korean story since the late 1960s (Kim & Jasanoff 2009).

To explore these early, alternative programs, we can track two developments. First, we look at the status of “radiation health” at the program’s inception, when the small group of Koreans affiliated with the institute was dominated by Japanese-trained physicists. As part of this discussion, we follow the growth of one of the two institutes affiliated with AERI, the RRIA (Radiation Research Institute for Agriculture) and the Radiation Research Institute for Medicine (RRIM), concentrating primarily on the second of these. Formed in the early to mid-1960s, these two related projects have a tendency to be overlooked, in part because they were reabsorbed under AERI’s scope in 1973 (Kim 2012).^3^ Second, we return to the state of the field of “health physics” in the late 1960s and early 1970s, when the number of Korean staff members devoted to the field was growing, with their career trajectories offering insight into the process of building a new field. Collectively, these developments suggest that for the first decade of its operations (1959–1969), the Korean atomic program had little to do with the pursuit of commercial power, and much more to do with the receipt and handling of isotopes in these diverse research contexts. Moreover, the Korean research community in this field held far more diversity than has been assumed previously, a reality obscured by the dominant America-centric narrative.

In this sense, the program reflects many of the original goals that were called for when it was first established. Moreover, the joint emphasis on medicine and agriculture suggests a focus very different from the public image of AERI after 1973, when it changed its name to KAERI (Korea Atomic Energy Research Institute), and consolidated the various research agendas under one heading. This more recent move is generally associated with the tensions of the Yushin state (1972–1979), during which President Park Chung Hee suspended the Korean constitution and assumed political power as his personal prerogative. At this point, KAERI had moved ahead with the pursuit of electricity-generation, and as we now know, South Korea was also in the midst of a military-led, uranium enrichment effort (1974) (Hong 2013; Kwon 2016; Kim 2012). These security concerns emerged only in the late 1960s, however, and do not reflect the relative diversity and corresponding freedom of action of the program’s first decade.

## External Actors and Technical Assistance (1959): Promoting Basic Research at AERI

### Competing Ambitions Clash

Prior to, and simultaneous with the introduction of “Atoms for Peace,” external parties brought radioisotopes to the Korean peninsula bearing a message of goodwill. In the war’s aftermath, medical relief work formed a significant part of rehabilitation that involved the participation of multiple nations. In the case of what became the National Medical Center (NMC), three nations—Sweden, Norway, and Denmark—formed a consortium, collectively gathered under the label “Scandinavian Project.”^4^ Denmark and Norway undertook medical assistance during the war, with Norway providing NORMASH (Mobile Army Surgical Hospital) units, and with Denmark docking a hospital ship, the *Jutlandia,* off the coast of Busan. The NMC project offered a chance to continue this wartime relief, now transitioning to postwar rebuilding. Other European nations participated in similar projects, with the West Germans operating a Red Cross hospital, thereby reinforcing the image of South Korea as a showcase development work (Hong 2015: see Chapter Three, “Mission Impossible,”: 83–109).

Taking the site of the Seoul City Hospital, the Scandinavian consortium sought to refurbish its physical facilities and in the process recast the location as the “National Medical Center.” In its design, the organizers envisioned a teaching hospital equipped with the latest equipment and practices from Europe (The National Medical Center in Korea 1971). Here is where radioisotopes entered, as a radiotherapy unit would be based within the hospital, offering the latest technology through a specialist form of cancer treatment. As the hospital and its programs existed under the aegis of a United Nations project (UNKRA, or United Nations Korea Reconstruction Agency), there was a lengthy period of discussion (1954–1958) concerning the contractual relationship, as well as the transport and handling of such sensitive materials. Priority represents less of an issue here (that is, which party was first in providing these materials), and rather, we must recognize that concerns surrounding radiation were already being negotiated in settings other than those under the domain of AERI: the emerging nuclear program did not hold an exclusive claim.

Following the war, the mid-1950s offered a diversity of sources through which such materials might arrive, and the regulations and expectations for their handling were by no means consistent. Prior to AERI (1959), South Korea had established its OAE (Office of Atomic Energy, 1956), which fell under the Ministry of Education (MOE). In turn, AERI came under OAE’s purview (MOE > OAE > AERI), meaning that the reactor site and its ambitions ultimately yielded to educational needs and a corresponding set of national goals (Kim 2012). Within this hierarchy, OAE held the power to grant or deny access to Korean scientists, as well as to provide funding and critical resources in a recovering nation. Not surprisingly, there was heated competition, and the ability to demonstrate expertise became the deciding factor in questions of influence and funding. In other words, those Koreans with prior experience, or those who had support networks to advance their claims, were more likely to succeed. Initially, this meant a hierarchy based upon seniority, dominated by those with previous Japanese training.

When foreign experts arrived in the mid 1950s, their advice tended to start from the post-war, ignoring this previous history. When the IAEA offered its assessment of the South Korean program, its team noted a lack of expertise with respect to the transport and handling of the materials it planned to distribute (IAEA 1960).^5^ This report anticipated the visit of a mobile laboratory (Spring 1960), and therefore offered a broad assessment in preparation for its arrival (IAEA 1960). This characterization should not be surprising, given the relatively small size of the Korean scientific community, and the added complications of a colonial legacy, followed by circumstances of national division (1948) and the Korean War (1950–1953). With a TRIGA Mark II reactor based at the engineering campus of Seoul National University set to go critical, IAEA advised that a tentative set of policies on radioactive materials be designed as a provisional measure, noting that the Korean authorities had yet to issue any regulations of their own.^6^ This advice came in the context of the university’s postwar recovery (1954–1960), which was ongoing, and involved numerous international partnerships, collectively aimed at rebuilding the post-colonial nation. The IAEA was more than willing to engage in this kind of outreach, going so far as to recommend the services of a health physics officer, given the absence of such a figure in the Korean context (IAEA 1960).

As with other post-colonial nations, South Korea started sending its students abroad for nuclear training, in this case, as early as 1955. When IAEA composed its report, therefore, it was safe to assume that there were students whose education was “in progress” for many of the areas deemed a necessity, even as they had yet to return home. During the site selection and construction phases (1957–1959), University of Michigan representatives brought in George Hoyt Whipple as a consultant, with a Rochester specialist aiding in the design of a health physics component for the nascent program.^7^ This project consisted of basic safeguards, such as the monitoring of dose levels through badges, the calibration of the instruments to detect and measure radiation levels, and the corresponding monitoring of levels throughout the facility. While these issues were concerns in 1959, the program proceeded at an incremental pace, so the issue was expressed as more of a long-term problem, rather than as an immediate one.

Collaboration with experts like Whipple while waiting for Koreans to finish their studies and return was not the only way to proceed; and in fact, an alternative approach formed a significant part of the response. A number of Koreans who had already gone abroad held positions in the United States and Europe, and it was possible to regard these individuals as forming an imagined collective. In the following decade, the Korean government would aggressively recruit such individuals to return home, offering higher salaries as an incentive. They might participate in a ROK project as Koreans, in other words, without desiring to return home, and they might remain secure in their status as expatriate experts for the moment. Joon Taik Han was one such individual, completing a degree in radiobiology at University of Kansas, and thus possessing skills of great value for the domestic context (Han 1958). Han did the majority of his publishing and academic work in English, but was given a consulting role with AERI in the late 1950s (AERI 1969). Han, and others like him, formed part of a transitional solution and represented a community that mediated between overseas Koreans and those based at home, illustrating the larger spectrum of possibilities for scientists as transnational migrants.

This observation holds true as well for international collaborators based in Seoul, especially during the period of reconstruction (1954–1960) during the later years of President Syngman Rhee (1948–1960). Medical and relief work brought in any number of international partners following the Korean War, and in some cases, the projects (and researchers) stayed on until the mid or late 1960s. This strategy proved to be of benefit to work with radioisotopes, where such relationships helped a great deal in easing concerns over a lack of experience. As mentioned previously, the National Medical Center, which was the product of a relationship between Seoul and its three Scandinavian partners, was identified by IAEA as the possible site for a program in radiation medicine, one that would take place through collaborative channels (IAEA 1960). Specifically, IAEA conceived of placing a radiotherapy department in the NMC, with a joint collaboration taking place between the hospital (under the auspices of the Scandinavian Medical Board), the OAE (Office of Atomic Energy), and the South Korean government.^8^

Such a collaborative arrangement was possible because South Korea was very much in transition. It needed the assistance of international institutions to make claims to legitimacy, and moreover, to continue to recover materially from the recent period of devastation. For medicine, IAEA envisioned placing a radiotherapy section at the NMC, with some thoughts towards an incremental transition to Korean control. In fact, this pattern roughly follows the trajectory of the NMC as a hospital, with Korean control of the facility coming at some point in the late 1960s (1968), although not without contestation (The National Medical Center in Korea 1971). At the same time, there was also a small amount of research activity with radioisotopes at the Seoul National University College of Medicine in the areas of physiology and pharmacology (IAEA 1960: 18).

From this description, it should be clear that Korean scientific activities were highly dispersed, rather than centralized, and tied to the preferences of individual researchers, rather than coordinated. This characterization has its roots in Japanese colonialism, which did not favor the interests of the colony, especially in terms of training Koreans, and in the unusual circumstances underlying the formation of Seoul National University, which lacked a central campus until 1975. Even in 1959, more than a decade after the university’s inception (1946), a foreign partner seeking to establish the foundations of a new program or specialty might easily encounter multiple recipient institutions as candidates.

It is possible to make a few baseline generalizations about the nascent Korean program. First, medicine and agriculture quickly emerged as priorities, much more so than energy or any comparable research agenda. Second, the issue of creating standard practices and metrics (specifically for the handling and processing of radioactive materials) was addressed almost entirely through cooperation with international partners, many of whom had arrived as part of relief missions that were not necessarily tied to nuclear science. In some cases, there was no set policy in the home countries of the experts, but they did not necessarily inform the Koreans of this fact. The unusual circumstances of post-Korean War recovery meant that any number of foreign experts were now based in Seoul for a wide variety of projects, allowing the IAEA’s mission to be tied to facilities such as the National Medical Center (The National Medical Center in Korea 1971). This approach was intended as a temporary solution, until a sufficient number of Korean trainees could return home. Third, a strict binary model of home versus abroad for Korean students and scientists does not capture the complex dynamics of the patterns of migration (and personal identity), as we have seen.^9^ In particular, graduate students completing degrees overseas might elect to take an intermediate position along a much broader spectrum, consulting on projects in Korea while maintaining careers abroad. These individuals often served as valuable links to strengthen international networks.

## Accumulating New Forms of Expertise: Diversifying AERI (1959–1966)

If the situation in 1960 was fluid and contingent upon collaboration with a number of international partners, by midway through the next decade AERI had become a more dominant force, becoming an emblem for the newly-acquired legitimacy of the Korean nuclear establishment. The enthusiasm for medicine and agriculture translated into active programs in each of these two areas, leading to the creation of spin-off institutes with independent research agendas. Created in 1963 and 1966 respectively, the RRIM (Radiation Research Institute Medicine, 1963) and the RRIA (Radiation Research Institute for Agriculture, 1966) placed the Korean atomic program on a trajectory to pursue a diverse cluster of programs, with its researchers spread out at different sites (KAERI 2009). The former looked primarily at the medical applications for radiation and moved towards developing the emerging field of health physics. In turn, the second institute focused on agriculture, with a special focus on the possibility of developing plant hybrids and new applications for agriculture.

These developments bore the fruits of the infrastructure sketched out earlier in the planning stages. With its ground-breaking taking place in 1959, the TRIGA Mark II reactor at AERI went critical in 1962, thereby giving the institute’s members cause for marking the occasion. The institute’s leadership experienced high turnover, going through several new directors within about a decade, but the key transition was a move away from the theoretical physics orientation of the initial group led by Park Chul-Chae, and a number of other Japanese-educated scholars, including a number of physicists and chemists, and in the direction of a more practical approach under incoming head Choi Hyungsub (KAERI 2009: 89–132). Although he also began his career with a Japanese education, Choi represented a transitional generation, acquiring his terminal degrees from American institutions.

At the national level, this style was reflected in the political transition to President Park Chung-hee in 1961, who favored an applied, industrial-style science as the means to achieve national renewal (Kim 2012). Although these changes cannot be read simply as the execution of top-down designs, scientists and engineers experienced a greater degree of accountability, and in turn, research opportunities and funding were available to those willing to make such an effort. With more of the returnees assuming leading positions, this period shows the origins of transition to an America-centric narrative centered around the research reactor.

### Radiation Medicine

The program sought, and began successfully producing, its own radioisotopes for domestic medical use within several years of the TRIGA Mark II reactor going critical, beginning with production of Iodine 131. Prior to this, the institute used radioisotopes it first received from external sources, slowly accumulating knowledge about their use in medical settings (KAERI 2009: 76).^10^ Later, the production of domestic sources continued with the TRIGA Mark III reactor, with this second facility going critical in 1972. At this early stage, the program remained under state control, and it would not be until about two decades later (early 1990s) that private industry emerged as a player, generating radioisotopes for commercial use, as in the radiopharmaceutical industry. In contrast, the early to mid-1960s was devoted primarily to uses such as teletherapy—directing radiation to treat diseased or cancerous tissue—and learning how to control and calibrate such practices (KAERI 2009: 76–88).

There was a precedent for this activity to be found in the colonial period, when, during the 1930s, radiation therapy was introduced to Keijo Imperial University Hospital, along with a handful of selected sites (KAERI 2009). In this limited setting, the therapy was directed at uterine cancer. At this time, and indeed, for a long time, cancer carried with it a powerful set of cultural taboos, and was generally not discussed publicly. In other words, the availability of treatment was limited on technical grounds, and further circumscribed by limitations on the circulation of information given a high degree of stigma. In its report from 1960, IAEA mentioned this pre-1945 context, but suggested that Koreans had little experience in the proper handling of materials (IAEA 1960: 18; Nelson 2017). For this period, and the immediate postwar era, this discussion was relevant in that it underscored the reality that most Koreans were unlikely to be familiar with, or to embrace, this style of treatment.

Keijo was not the colonial site to explore the possibility of radiation therapy, and Severance Hospital, an institution founded through overseas mission resources, also explored radiation’s potential in medicine, albeit under highly specific conditions. The radiation oncology department at Severance reported the use of Japanese technology in the late 1930s (1937), using X‑rays primarily for diagnosis and treatment of cancer (Huh & Kim 2020). With Japanese technology appearing again following 1965, this time it received sponsorship from OTCA (Overseas Technology Cooperation Agency), the predecessor to JICA (Japan International Cooperation Agency) installing radiation therapy equipment at Severance in the early 1970s (Huh & Kim 2020).

The same body of literature referencing the device at Severance during the colonial period also notes uses of x‑ray technology in the period 1953–1960, in the immediate aftermath of the Korean War. The OTCA-sponsored device next arrived in 1972, marking the beginning of more ambitious attempts at cancer treatment, with more aggressive targeting for therapy (Huh & Kim 2020). This technology from Japan was more widespread in the post-1965 period, as Korean public health campaigns at the time relied heavily on Japanese-manufactured goods. For example, the anti-parasite campaigns (1969—early 1990s) used Japanese microscopes, slides for processing samples, and even the curriculum to train the technicians (Homei & DiMoia 2021). In some cases, Korean doctors and scientists re-established contact with their Japanese colleagues from two decades earlier, especially in the biomedical sciences (Homei & DiMoia 2021). If there was tension over colonial history, there was also recognition that shared knowledge and opportunities should take priority.

In constructing a post-war story, most larger hospitals in Korea date their radiation therapy programs only to the early to mid-1980s, a period associated with much greater wealth, and with a much larger medical community prepared to integrate the new technologies. When such programs were first announced in the early 1960s, this gesture often mean that work was to be done on a trial basis, certainly not with a significant pool of patients.^11^ Under these restricted conditions, Korean medical facilities were operating cobalt treatment equipment as early as 1963, again emphasizing an exploratory program geared to learning how to operate the devices. More frequent use, and the corresponding act of familiarizing patients with such forms of cancer treatment, would have to wait until the early 1980s. The issue of cost also forms a key part of the picture, as health insurance, although it existed in theory from the early 1960s (1963), did not come to resemble its more recent (national) form until the reforms associated with democratization in the late 1980s (Wong 2006).

Given the relative lack of commercial uses, the field of health physics was generally restricted to two specific sites: hospitals with radiation therapy units and the AERI site itself. For the former, the ability to control radiation for therapeutic uses was an important goal, as was the related task of limiting the effects of radiation beyond the desired target. Even with these priorities, only a few employees at AERI were devoted to field: in total, they numbered numbering less than ten before the nuclear industry in South Korea took off in the 1970s. Trainees sent abroad in the initial wave of enthusiasm were beginning to return home, and the AERI staff grew in a corresponding fashion, providing jobs for these valued individuals. By 1969, among a staff of more than forty, six individuals were based in the health physics section (AERI 1969).

AERI appointees tended to hold degrees with training more specific to their jobs at a nuclear center. In contrast, those working elsewhere with radiation medicine in health facilities were often M.D. trainees, with some further training tailored to the technology itself (M.C. Lee et al. 2010). Although nominally sharing the same field, the two clusters represented very different types of experts, and to this point, the training had much more to do with defining and approaching cancer in the Korean context. AERI’s institutional dominance in characterizing its role working with radiation has a lot to do with its role as a small, powerful community, one able to promote its story within the historiography. Defining “health physics” more broadly shows that this community was actually diverse in both their training and their approaches.

## Crafting Networks of Expertise, 1966–1969

The second main strand of health physics examines those individuals more closely associated with AERI, whether over the long term or for a short-term affiliation. To review, these individuals were among the first generation to study abroad, as they took up graduate work at a range of American and European institutions. The prominence of post-war American science, along with South Korea’s special relationship with the United States, has produced a popular narrative in which this period reflects the “Americanization” of the field. At an even broader level, Professors Kim Geun-bae and Moon Man-yong of JBNU (Jeonbuk National University) have led a series of debates concerning the relative contributions of Americans and Koreans to postwar science, especially in the new institutions such as KIST (Korea Institute of Science and Technology) (Moon 2021). Yet it is far from clear that American influence held a monopoly over developments.

Before 1980, health physics primarily referred to the uses of radiation health, especially in terms of early forms of cancer treatment. In part because of the stigma associated with cancer, and the challenge of learning these new technologies, radiotherapy did not achieve fruition until the early 1980s, when it became available to patients in sufficient numbers (Nelson 2017; Suh 2016). Prior to this, AERI dominated the narrative as a national institution, and the goal here has been to show that activities conducted outside of AERI offer a wider perspective on the field’s development. As we return the focus to AERI, even newcomers within the institution were beginning to change the internal culture.

Along with their research activities, the travels of these researchers as both practitioners and scholars offer insights into the broader trends of development in Korean health physics. The first generation of trainees, trained in the late 1950s, typically had some degree of participation in the Japanese system, and were fortunate enough to continue their training with American assistance, whether through a short-term course, such as at Oak Ridge, or via a full-scale nuclear engineering curriculum (Penn State, NC State, UC Berkeley) (North Carolina State University Nuclear Engineering).^12^ By the late 1960s, this pattern had diversified, to reflect changes not just to the Korean program, but to other actors, especially those in Europe and Japan. A number of Koreans had acquired experience in the Japanese system (AERI 1969). By the mid to late 1960s, Japan had become one of the leading donor nations for East Asia, and in particular, its technical training programs were important to neighbors in the region (Mizuno et al. 2018).

The head of the health physics section of AERI, Rho Chae-Sik, held his degrees from Seoul National University (B.S., Ph. D) and Imperial College, with his overseas training taking place in Norway as a visiting researcher. Rho’s appointment at age 39 made him relatively young, and indeed, the general trend among this group of researchers was their youth, with the ages ranging from about the mid-thirties to early forties. In addition, Rho was one of only two Ph.Ds in the section, with the four remaining members holding either a Bachelor’s or the combination of a Bachelor’s and Master’s. Military experience was also a common denominator, which was no surprise for a nation with required conscription, and further service likely indicated the value placed upon their skills by the Army and Air Force. Three of the six had scientific training in Japan, and this factor along with a corresponding number of IAEA placements and training courses, represents probably the single most significant development in terms of building up emerging networks of expertise (AERI 1969; Pike 1972).

Rho’s career trajectory serves to underscore developments representative of the larger community, as he remains best known for his work on climate and the effects of micro-pollutants (National Academy of Science). As a “late developing” country, South Korea avidly pursed its industrialization and growth for nearly three decades (from the early 1960s through the mid-1980s) before questions about the effects of these policies emerged in the early 1990s. A scientist like Rho used his knowledge of radiation to manage and watch for its presence in the surrounding environment, and extrapolated from these observations to consider the wider range of potential health hazards. While this knowledge might have been applied within the nuclear industry, it also reflects a broader embrace of a vision critical of, and careful with, any wholescale use of applied science.

Rho’s career stands out as significant not only for what it indicates in terms of building networks, but also for its emphasis on a pragmatic vision, one perhaps distinct from the typical narrative for “health physics.” Whereas the category tends to be associated with nuclear programs specifically, Rho sought to apply his expertise more broadly, covering any of a range of pollutants that might affect the South Korean population. In this sense, he, and others of this generation of Korean specialists, chose to construct a different category, one connected to environmental science, and which might be best characterized as “environmental health.” Again, for a specialist in atmospheric science, Rho approached a range of topics, and this observation holds true for many others in a small country. This situation led him to pitch his research more broadly, so that it might cover multiple jobs or tasks in order to respond to acute needs in a rapidly changing country with a shortage of trained personnel.

For his research, Rho worked individually and with others, with his publications indicating the direction of his developing interests. A 1970 article considered the impact of environmental radiation, specifically naming fall-out from China as the object of inquiry (Rho 1970). After demonstrating its nuclear capabilities through a bomb test in October 1964, China forced its neighbors to reckon with it as a nuclear power: South Koreans were acutely aware of this danger due to its proximity. In fact, the majority of Rho’s early publications refer to fall-out and the environmental effects of radiation (Rho 1963, 1967), a cluster with both regional and domestic implications. Over the course of his career, these interests would broaden to include wider questions of environmental engineering and Korean development (Kim 2022; Kim et al. 2022).

In view of these developments, situating the narrative of a South Korean program within a US regulatory nexus proves limiting, and the meaning of “health physics” takes on a modified role. In terms of its own institutional bodies, South Korea proceeded to form KARP (Korea Association for Radiation Protection) in 1974, with this advisory body standing alongside the growth of a commercial nuclear industry in the late 1970s. In this context, Rho, and others like him, gained access to tremendous opportunities for career growth, but they also worked within constraints specific to Korea under military rule. In this setting, “radiation protection” took on a wider set of meanings, and it may prove useful to consider whether these multiple tasks aided, or hindered, any of the powers granted to KARP as a new type of nuclear industry watchdog.

## Beyond Institutional History: Korean Health Physics, Japan, and Regional Exchange

Korea’s technical exchange relationship with Japan never entirely lapsed. The period between 1945 and 1965 represented a reconfiguring of the relationship as the two countries negotiated how they might collaborate under new circumstances. As we have seen, the first generation of Korean elites running government programs typically had received Japanese training, and the post-war period allowed for a reconsideration of how to work together again. These questions were not only restricted to South Korea’s relationship with Japan, as it sought to normalize its relations with numerous partner nations in East and Southeast Asia.

The American-influenced narrative of AERI development offers an idealized vision of progress in the orderly control of radiation and smooth succession (from the Japan-trained generation to the overseas or “American” generation), omitting much of the political contestation and anxieties over regional radioactive fallout. In fact, fears of fallout lingered long after 1945, and were expressed in the form of visual art, theater, and related forms of representation. These concerns appeared anew during the Korean War, with the prospect of the bomb’s use; and again in 1964, when China developed nuclear capabilities. Newer scholarship evaluates these issues both from a cultural perspective and from an STS point of view (Kramer 2021). Nonetheless, it is important to note that activities concerning radiation did not always fall under the purview of AERI, but also came through the efforts of individuals working in a cluster of distinct fields, as we have seen here with the case of health physics.

Along these lines, Derek Kramer tracks these newer forms of expression in “A New Kind of Energy,” which looks at the two Koreas and a variety of writings on radiation (Kramer 2021). In one prominent example, Kramer cites an agricultural study conducted at Kyungbuk University in the late 1950s, where nearby fields were used as the primary data set (Kramer 2021). Several experimental fields were used to grow pumpkins, eggplants, and peppers, with the end products then gathered for evaluation. Using local precipitation and meteorological data, along with data from the exposed plants, the university’s scientists built a picture of fallout effects for Korean villagers. This type of activity defined health physics in a way that AERI expressed in more cautious terms, shying away from examining fallout’s direct effects.

The goal here has not been to dismantle or minimize the AERI narrative, but rather to probe its limits. While traditional narratives characterize Korean nuclear science as characterized primarily by the simple transfer of colonial (Japanese) models of expertise followed by a postwar turn to American influence, the focus on health physics reveals a more complex picture. Instead of seeing AERI’s role as a governing institution as one characterized by a culture of regulation and control, we see how such notions remained very much open to question.

### Regional Technical Training: East Asia

Along with the goal of seeking a wider context for radiation, the secondary goal here has been to resituate Korea within its regional context, instead of relying exclusively on American/international comparisons. These types of metrics, comparing new forms to American or European standards, became enormously popular in the post-Korean War Period, when the goal of recovery required such standard-setting. However, as we have seen, much of the research activity continued to be domestic and regionally focused, with legitimate fears tied to nuclear activities taking place nearby (for example in China in 1964 and India in 1974). The story of the post-1965 normalization of relations with Japan continues this theme, as we see Korea articulate a new, post-colonial identity.

For radiation and agriculture specifically, Japan’s role as a leader in agriculture and in breeding experiments served as one of these new points of contact (Kim 2018). The rice paddies of Hiratsuka (near Tokyo) served as one of the agricultural stations where cross-breeding experiments might take place irrespective of their irradiation. As a result, many Korean agronomists sought to spend time working in Hiratsuka to benefit from the prior experience of their colleagues. In terms of the larger perspective, this type of network-building activity involved both “push” (top-down planning and coordination) and “pull” (specialist interest and the maintenance of personal networks or relationships) factors. The key element, in short, lay in the emergence of a regional partner, and a collaborative relationship that did not involve the direct participation of the United States.

Along with the site at Hiratsuka, Ibaraki Prefecture served as a home for the IRB (Institute for Radiation Breeding), which became a Japanese national institute in 1960. The IRB hosted visitors from any number of regional partners, not just Korea, and promoted Japan’s role as a leader in agriculture and mutation breeding. Within the field of medicine, collaboration began in 1968, with Japan’s OTCA (Overseas Technical Cooperation) supplying a great deal of equipment and training to Korea prior to the introduction of the Korean National Anti-Parasite Effort in 1969. The approach to normalizing relations with Japan was comparable to those made with other developing countries and served as an attempt to minimize tensions (Homei & DiMoia 2021).

Rho, along with several of his colleagues with similar career trajectories, formed a core part of the health physics section at AERI through the late 1960s (1960–1973), with a shift coming early in the following decade. If the term “health physics” had initially held an indistinct meaning in the early 1960s, by 1974 it had become clarified in both institutional and linguistic terms. With Rho as a prominent member, scientists formed KARP (Korean Association for Radiation Protection), an organization which continues to operate in the present day. The organization’s website indicates that the choice of language—“radiation protection” in KARP—was very much intentional, reflecting not so much scientific practice and instead fit within regional and local politics (KARP). The term “health physics” was widely used in Japan, and the new terminology indicates a Korean effort to develop its own brand of identity focused instead on radiation protection.

Japanese scientific expertise and collaboration became critical to Korean science and technology and provided a close regional partner and an alternative source of external validation to the United States. To repeat a point made earlier, three of the six Korean members of KARP, with Rho prominent among them, had experience in Japan as visiting scholars (AERI 1969). In contrast to the colonial period, working with, and publishing under these conditions, now conferred recognition of Korean capabilities and contributions. If the Korean version of “radiation protection” was not simply a case of language, and aimed to become its own field, it needed to find itself, and this strategy was part of the scheme that characterized an emerging, post-colonial Korean scientific establishment.

## Footnotes

1 “Joint Explanatory Declaration” (June 12, 1961), Folder S‑0526-0094-0005—National Medical Center in Korea (UNARMS, United Nations Archives and Records Management Section).

2 Meinke, Wayne “Report on Visit to Korea (April 1962),” Box 24, 4–5, Michigan Memorial Phoenix Project Papers, Bentley Historical Library (BHL), University of Michigan.

3 The new name, KAERI (Korea Atomic Energy Research Institute) reflects the transition to nuclear engineers (and away from physicists), and its consolidation as a single body.

4 “Joint Explanatory Declaration.”.

5 This report covers a broad slice of East and Southeast Asia: Japan, Taiwan, Korea, the Philippines, and Vietnam.

6 This language echoes that of the Phoenix Project materials (University of Michigan) for the reactor. This serves as an example of how visiting experts typically recommended the adoption of external rubrics.

7 Box 22, “Korea 1958–1959,” Michigan Memorial Phoenix Project. University of Michigan, Bentley Historical Library (BHL).

8 See National Medical Center in Korea (1971): 87–94 for a statistical portrait of the NMC, including the relevant figures for X‑rays and use of teletherapy. For a longer view of the history of the NMC, documents concerning its origins may be found at the UN Archive (UNARMS) in New York within the UNKRA files.

9 This observation holds true for many fields, as the ROK government under President Park (1961–1979) frequently contacted overseas Koreans for assistance.

10 Lee Moon-ho tried to import isotopes from West Germany in 1959, only to discover that there were no procedures for their shipping.

11 In addition to the NMC, other major hospitals also had formative programs.

12 Raymond Murray at North Carolina State (NCSU) operated one of the earliest training programs.
